# Hepatoprotective effects of almond shells against carbon tetrachloride induced liver injury in albino rats

**DOI:** 10.1016/j.sjbs.2023.103811

**Published:** 2023-09-14

**Authors:** Andleeb Zahira, Salma Sultana, Azhar Rasul, Tayyaba Sultana, Mudassir Hassan

**Affiliations:** Department of Zoology, Government College University Faisalabad, 38000, Faisalabad, Pakistan

**Keywords:** Antioxidants, Hepatotoxicity, *In vivo*, Almond extract, Phytochemical

## Abstract

Liver injury is a prevalent pathological process that can give rise to conditions such as fatty liver, cirrhosis, fibrosis, and even cancer. It has been observed that plants and natural products possess significant protective effects against liver injury. Current study was performed to investigate the efficacy of almonds shell against carbon tetrachloride (CCl_4_) induced hepatotoxicity in rat model. As almonds shell contain a large variety of phenolic and flavonoid compounds, which are largely associated with antioxidant and hepatoprotective properties. For this purpose, screening of small-scale library of twenty plant extracts was performed for evaluation of antioxidant potential by DPPH assay. The data revealed that almonds shell extract (ASEE) exhibited potent antioxidant activity. This potent extract was further evaluated for hepatoprotective activity in *in vivo* rat model on 30 rats, divided into 6 groups of 5 rats each. On 29th day all rats were sacrificed and blood serum was collected for further analysis. Liver tissues were also preserved in formalin for histopathology. The results demonstrated that ASEE displayed a protective effect on liver function tests (LFT), renal function tests (RFT), and lipid biomarkers in comparison to the CCl4 group. The histological data also unveiled a substantial safeguarding impact on liver damage, characterized by a reduction in apoptosis, diminished liver hemorrhage, and decreased accumulation of cellular debris. The data indicates that ethanolic extract from almond shells possesses hepatoprotective potential, suggesting its viability as an alternative source for hepatoprotective drug development after pre-clinical research.

## 1 Introduction

Oxidation is an essential process for the production of energy, required for cellular activities. Unavoidable free radicles are produced during oxygen metabolism, commonly called reactive oxygen species, responsible for many diseases (Chidambara Murthy et al., 2002). Unsaturated fatty acids are attacked by these radicals, resulting in peroxidation of lipids and the destruction of DNA and proteins. As a result, a chain of harmful changes starts in the body which ultimately causes cell inactivation. These radicals are responsible for many malformations of the liver. Hepatic disease is mainly due to biological agents such as viruses, bacteria and parasites and different chemicals like dimethyl nitrosamine, carbon tetrachloride, over use of alcohol, and thioacetamide (Morencos et al., 2008). However, antioxidants play an important role which can stop the production of free radicals, protect from ROS in biological systems and maintain the internal redox potential (Unsal et al., 2020). These antioxidants react with these free radicles and raise the defense system by expressing genes that are involved in the production of catalase, superoxide dismutase and glutathione peroxidase. The liver plays a key role in the maintenance of many physiological functions like metabolism, storage and secretion. Its ability to detoxify waste and toxic compounds and formation of useful agents, is well known (Sobeh et al., 2020).

Wasted parts of fruits and vegetables such as peels and seeds contain minerals, vitamins and phenols, which are good antioxidants that play a key role in removal of free radicals and thus are useful in the protection of the body from many diseases (Mosa and Khalil 2015). More than 100 plant species have been reported which contains 160 phytochemicals that have hepatoprotective effect (Ali et al., 2019). Many modern medicines are available but only a few natural medicines are found to be effective in liver treatment. These medicines increase the efficacy of the liver, protect from harmful agents and repair the damaged hepatic cells (Maqbool et al., 2019).

A wide range of phenols and flavonoids are found in almonds which increase their antioxidant activities. A major portion of almonds consists of fat and protein (Rao and research, 2012, Xie and Roto, 2012). Its fatty acid composition is helps lower the LDL cholesterol. Its use is helpful as a cerebrotonic, cardiotonic, deobstruent of the spleen and liver, laxative, antitussive and aphrodisiac drug (Abdullah and Hussain 2017). Three Important triterpenoids such as betulinic, oleanolic acids, betulinic (Takeoka et al., 2000) and flavonol glycosides (Sang et al., 2002) are present in high concentration and other compounds protocatechuic acid, p-hydroybenzoic acid, vanillic acid, glucoside, 3B-O-methylquercetin are also reported (Sang et al., 2002). Almond contains iron and copper so, found to be helpful for anaemia (Sahib 2014). Almonds shell have Xylose-oligosaccharides which are xylose-based oligomers, are non-carcinogenic and have prebiotics potential in the colon (Yuan et al., 2005), also lowers the risk of colon cancer (Wollowski and Rechkemmer, 2001, Hsu et al., 2004). However, hepatoprotective effect of almond shells was still unknown. So, present study was conducted to find the hepatoprotective effect of almond shells by feeding the Wistar albino rats against CCL_4_ by histopathological and biochemical methods. Lipid peroxidation, catalase and peroxidase in liver homogenate were estimated to determine the protective effect provided by almond shells against carbon tetrachloride.

## 2 Materials and methods

### 2.1 Collection and extract preparation of food wastes

Twenty selected food wastes were collected, cleaned and dried at normal room temperature. After two weeks, ground them into a coarse powder. The extracts were prepared by using green extraction technology. Two hundred grams of food waste powder was taken in a flask and 300 ml of ethanol was added into it, then the mixture was heated for 6 mints in a microwave oven and the liquid was filtered by using Whatman 1 filter paper. This process was repeated five times with the same sample and then filtrates were concentrated by using vacuum rotary evaporator, to remove the solvent and make the extract concentrated. On solidifying the extracts, a number was assigned and recorded in extract library. Plant parts used in the research are listed in Table 1.

**Table 1:** Characteristics of selected food wastes.

Common names	English names	Botanical names	Parts used
Meethy	persian lime	Citrus latifolia	Peels
Halwa kaddu	Pumpkin	Cucurbita moschata	Peels
Halwa kaddu	pumpkin	Cucurbita moschata	Seeds
Saib	Apple peels	Malus domestica	Peels
Green gram	Mung	Vigna radiata	Husk
Badam	Almons	Prunus dulcis	Shells
Akhrot	Walnut	Juglans regia	Shells
Mosambi	Sweet lime	Citrus limetta	Peels
Pyaz	Onion	Allium cepa	Peels
Malta	Orange	Citrus sinesis	Peels
Chakotra	Grape fruit	Citrus paradisa	Peels
Phaliyan	Beans	Phaseolus vulgaris	Peels
Amrood	Guava	Psidium guajava	Peels
Palak	Spinach	Spinacia oleracea	Peels
Aam	Mango	Mangifera indica	Seed coat
Lahsun	Garlic	Allium sativum	Peels
Arvi	Taro	Colocasia esculenta	Peels
Kaylaa	Banana	Musa acuminate	Peels
Garma	Cantaloupe	Cucumis melo	Peels
Matar	Pea	Pisum sativum	Pods

### 2.2 Antioxidant activity by DPPH assay

The antioxidant activity of ethanolic extracts of all these twenty mentioned extracts were determined by using DPPH assay by the method reported by Zhang et al. with modifications (Zhang et al., 2013). Initially, all the extracts were screened at a single dose 250 µg/ml and potent extracts were identified which further validated with multiple doses. To prepare DPPH solution, 2 mg of DPPH was dissolved in 50 ml of methanol. Antioxidant activity of almond shells ethanolic extract was determined at different concentrations (62.5 µg, 31.25 µg, 15.62 µg, 7.6 µg, 3.8 µg and 1.9 µg) in 96 well plates at 517 nm. Ascorbic acid and methanol were used as control and blank respectively. Antioxidant percentage was calculated by following formula.

$$\text{\textbackslash{}\% age of Antioxidant = 1}-\frac{\text{Sample Abs}\;}{\text{Control Abs}\;}\;\text{x 100}$$

Here, Abs stands for Absorbance.

### 2.3 Animal acclimatization

The determination of *in vivo* antihepatotoxicity of almond shell extracts was done by following the protocol explained by Hussain et al., with possible modifications (Hussain et al., 2022). For this purpose, 30 Wister rats having 150–200 g weight were taken from the animal house of the Department of Zoology, Government College University Faisalabad. Standard laboratory conditions were provided to the animals, with 12-hours day & night cycles, free access to water and standard diet, temperature (24 ± 2) and humidity (55 ± 5%) was maintained. For acclimatization purposes, the animals were kept in maintained conditions for a period of one week. The experimental protocol was authorized by Government College University Faisalabad’s Ethics Review Committee.

### 2.4 Experimental design

To determine the hepatoprotective activity of almond shells ethanolic extract against carbon tetrachloride, the following research experiment was performed for the duration of 28 days.

6 uniform groups of rats were formed.

Group 1.

Rats were treated with distilled water by oral gavage.

Group 2.

Rats were treated with oral administration of CCl_4_ (2 ml/Kg) after every 7 days during the trial to induce liver toxicity.

Group 3.

Rats were treated with silymarin for 28 days with a gap of 24 h; the animals were intoxicated with CCl_4_ after every 7 days.

Group 4.

Almond shells ethanolic extract (200 mg/kg Body weight) was given for 28 days with the gap of 24 h and the rats were treated with CCl_4_ after every 7 days.

Group 5.

Almond shells ethanolic extract (400 mg/kg Body weight) was given for 28 days and CCl_4_ treatment after every 7 days.

Group 6.

Almond shells ethanolic extract (600 mg/kg Body weight) was given for 28 days and CCl_4_ treatment after every 7 days.

Distilled water was used to dissolve the extract and suspension was made in CMC. 3 ml/kg of CCl_4_ was given to groups 2, 3, 4, 5 and 6.

### 2.5 Sample collection

Rats were sacrificed after 24hrs of the last treatment of CCl_4_ by cutting at cervical region. Blood was collected in purple and red cap bottles. Purple cap bottles were inverted about 6–8 times to mix the blood with EDTA, to prevent the blood from coagulation. Red cap bottles were placed vertically for about 15 min to separate the serum from blood. Blood was centrifuged for 15 min at 3000 rpm. Supernatant was then separated and stored into eppendrof tubes. For the examination of histopathology, liver of rats was preserved in 10% formalin.

### 2.6 Liver function tests (LFT)

Liver enzymes aspartate transaminase (AST), alanine transaminase (ALT) and alkaline phosphatase (ALP) was measured in the serum by the use of standard protocols provided by SPECTRUM ALP/GPT kit, INNOLINE ALP kit and SPECTRUM AST/GOT diagnostic kits and absorbance was calculated by semi-automatic MicroLab 300 biochemistry analyzer.

### 2.7 Renal function tests and lipid profiles

Level of urea, creatinine, cholesterol and triglyceride from stored serum were determined by following standard protocols mentioned in their respective kits purchased from SPECTRUM and results were taken by using biochemistry analyzer MicroLab 300 EXL.

### 2.8 Assays for hematological parameters

Blood in EDTA tubes were used for complete blood count (CBC) to count the hemoglobin level, red (RBCs) and white blood cells (WBCs) number was measured by Siemens hematology analyzer.

### 2.9 Histopathology

Liver preserved in formalin was cut by using sharp blade, and a piece of tissue was kept in histology cassettes. Fixation of tissue was done in formalin and dehydrate these tissues in 60%, 70%, 80%, 90% and 100% ethanol, then embedded in wax. Tissues were cut into thin sections of about 5um by use of microtome, transferred them on glass slides covered with egg albumin. Staining was done with hematoxylin and eosin. Liver tissue was observed by using optical microscope and images were taken with camera.

### 2.10 Statistical analysis

IBM SPSS statistics was used to analyze the data. One way ANOVA was used to compare the data between the groups to evaluate variations and significance. After analysis, data was represented by mean ± standard deviation. The results exhibit p < 0.05, results were considered as significant.

## 3 Results

### 3.1 Screening of 20 selected food waste extracts for antioxidant activity

**Fig. 1:**
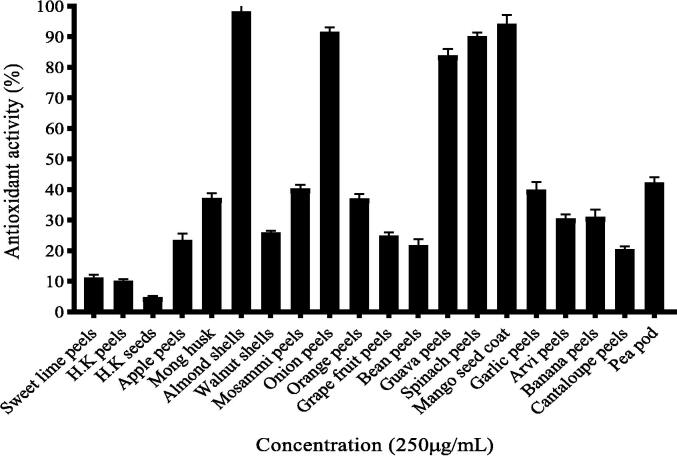
Antioxidant activity of food waste extracts at 250 µg/mL.

Ethanolic extracts of 20 selected food waste were screened for antioxidant activity through DPPH assay, using ascorbic acid as standard. Almond shells (*Prunus dulcis*) show the maximum antioxidant activity following in the descending order by *Mangifera indica* (Mango*), Allium cepa* (Onion), *Spinacia oleracea (*spinach*), Psidium guajava, Allium sativum, Citrus limetta, Pisum sativum, Vigna radiate, Citrus sinensis, Musa acuminate, Colocasia esculenta, Citrus paradise, Juglans regia, Malus domestica, Phaseolus vulgaris, Cucumis melo, Citrus latifolia, Cucurbita moschata* (peels), *Cucurbita moschata* (seeds) as shown in Fig. 1.

### 3.2 Dose dependent antioxidant activity of almonds shell extract

Antioxidant activity of almond shells was checked at different concentrations 1.96 µg/ml, 3.9 µg/ml, 7.87 µg/ml, 15.75 µg/ml, 31.25 µg/ml and 62.5 µg/ml. The calculated IC50 of almond shell was determined at 15.4 µg/ml which was less than standard ascorbic acid 31.15 µg/ml which indicating the antioxidant potential of ASEE is significantly higher (P < 0.001) than standard ascorbic acid as shown in Fig. 2a & Fig. 2b.

**Fig. 2a:**
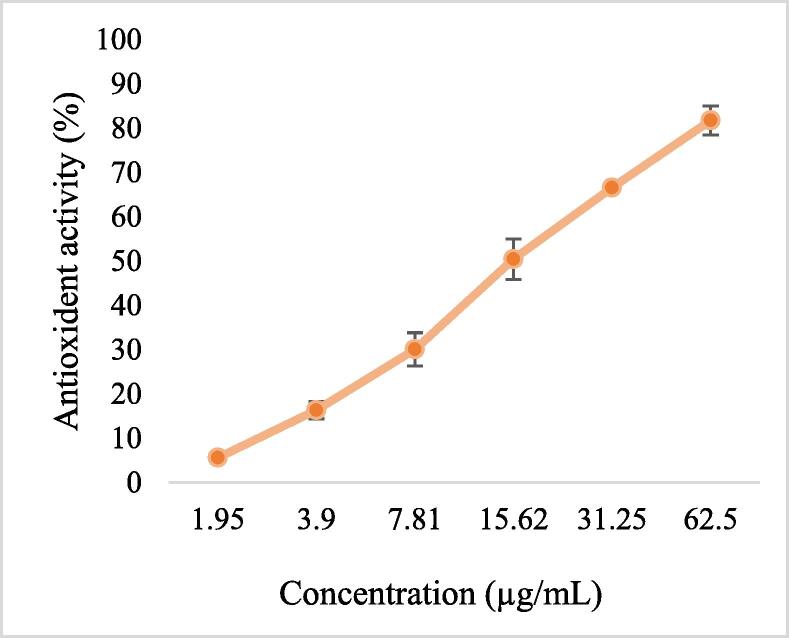
Antioxidant activity of ASEE.

**Fig. 2b:**
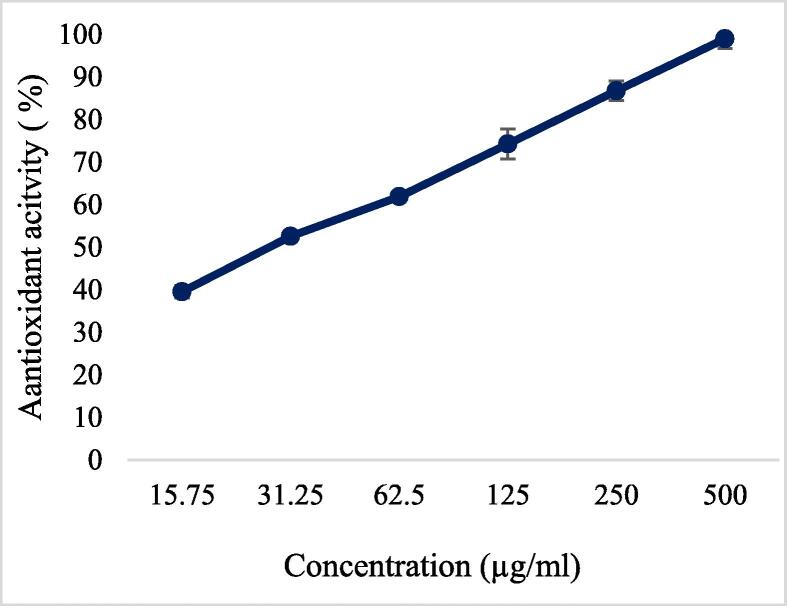
Antioxidant activity of ascorbic acid.

### 3.3 Effect of almond shells ethanolic extract on LFTs in CCl_4_ intoxicated rats

CCl_4_ administration significantly (p < 0.001) increases the level of ALP, ALT and AST in serum samples of rats as compared to control group (460 ± 26.98 U/L, 340 ± 13.91 U/L & 388 ± 13.93 U/L Vs 394 ± 15.89 U/L, 272 ± 19.35 U/L & 186 ± 17.86 U/L respectively). In ASEE administrated rats with ethanolic extracts of almond shell (200 mg/kg, 400 mg/kg & 600 mg/kg) significantly lower the level of AST (359 ± 24.04 U/L, 348 ± 23.93 U/L & 202 ± 11.57 U/L), ALT (320 ± 6.36 U/L, 252 ± 10.59 &185 ± 20.33) and ALP level was initially decreased at 200 mg/mL but little increased at 400 mg/kg and again lowered at 600 mg/kg indicating non significance in ALP reduction as shown in Table 2.

**Table 2:** Effect of ASEE on liver function parameters (LFTs) of CCl_4_ damaged livers in rats.

Group	Treatment	AST (U/L)	ALT (U/L)	ALP (U/L
Group Ⅰ	Negative Control	186 ± 17.86	272 ± 19.35	394 ± 15.89
Group Ⅱ	CCl_4_ Treated	388 ± 13.93**	340 ± 13.91**	460 ± 26.98**
Group Ⅲ	Silymarin & CCl_4_ Treated	111 ± 24.58	229 ± 8.24	373 ± 19.22
Group Ⅳ	200 mg/kg ASEE & CCl_4_ treated	359 ± 24.04	320 ± 6.36	355 ± 17.67
Group Ⅴ	400 mg/kg ASEE & CCl_4_ treated	348 ± 23.93	252 ± 10.59	481 ± 6.11
Group Ⅵ	600 mg/kg ASEE & CCl_4_ treated	202 ± 11.57**	185 ± 20.33**	458 ± 19.60*

ALT: Alanine transaminase, AST: Aspartate transaminase, ALP: Alkaline phosphatase. Where *p < 0.05, **p < 0.001 and ***p < 0.0001.

### 3.4 Effect of almond shells ethanolic extract on lipid profile of serum in CCl_4_ toxicity induced rats

CCl_4_ treated rats showed significantly lower levels of triglycerides as compared to the control (43.2 ± 6.72 mg/dl vs 72 ± 6.08 mg/dl respectively). Almond Shell ethanolic extract (ASEE) and silymarin-treated rats showed considerable a increase in triglyceride levels (Table 3). ASEE showed a significant increase in the level of triglycerides at a medium dose (400 mg/kg) which was equal to silymarin (50.6 ± 10.2 mg/dl & 50.2 ± 13 mg/dl). A medium dose of ASEE (400 mg/kg) showed a significant recovery of cholesterol level in treated groups as compared to CCl_4_ intoxicated rats in cholesterol level (28.25 ± 5.01 mg/dl Vs 35.85 ± 4.20 mg/dl). While 600 mg/kg dose showed slightly increase in cholesterol level this maybe due to accumulation of high proportion of monosaturated fatty acids and poly unsaturated fatty acids present in almond shells. Therefore, dose 400 mg/kg may consider best dose for effective reduction of cholesterol level in CCl_4_ intoxicated rats.

**Table 3:** Effect of ASEE on cholesterol and triglycerides of CCl_4_ treated rats.

**Parameters**	**Group I**	**Group II**	**Group III**	**Group IV**	**Group V**	**Group VI**
Cholesterol (mg/dl)	22 ± 4.84	35.85 ± 4.20	25 ± 7.03	29 ± 8.74	28.25 ± 5.01**	40 ± 7.54*
Triglycerides (mg/dl)	72 ± 6.08	43.2 ± 6.72	50.2 ± 13	45.5 ± 9.19	50.6 ± 10.2	42.25 ± 8.3*

Where *p < 0.05, **p < 0.001 and ***p < 0.0001.

### 3.5 Effect of ASEE on renal function parameters in CCl_4_ intoxicated rats

CCl_4_ intoxicated rats showed a significant increase in urea and creatinine levels. ASEE and silymarin decreased the level of urea and significant reduction was observed at 600 mg/kg conc. of ASEE (32.75 ± 5.67). Creatinine level was also observed significantly recovered by ASEE and silymarin except group V (400 mg/mL) where this trend was found non-significant Table 4.

**Table 4:** Effect of Almond shells ethanolic extract on renal parameters of CCl_4_ damaged livers in rats.

Parameters	Group Ⅰ	Group Ⅱ	Group Ⅲ	Group Ⅳ	Group Ⅴ	Group Ⅵ
Urea(U/L)	34.25 ± 7.4	48.2 ± 5.26**	38.5 ± 6.65	29.33 ± 4.9	32 ± 6.55**	32.75 ± 5.67**
Creatinine (mg/dl)	0.49 ± 0.1	0.76 ± 0.11	0.5 ± 0.06	0.46 ± 0.12	0.8 ± 0.1*	0.625 ± 0.13

Where *p < 0.05, **p < 0.001 and ***p < 0.0001.

### 3.6 Effect of almond shells extract on hematological parameters in carbon tetrachloride intoxicated rats

**Fig. 3:**
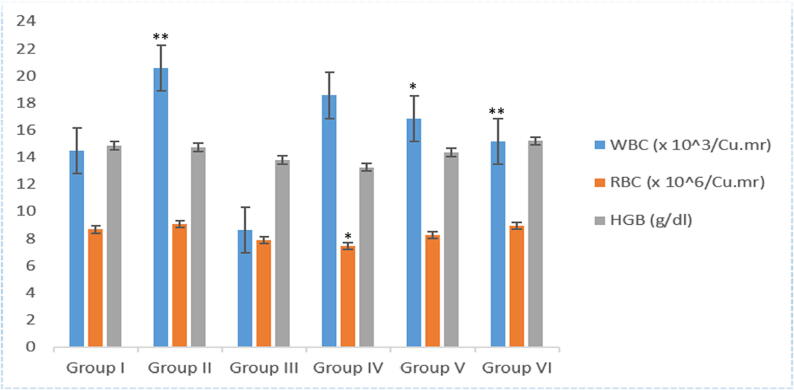
Effect of Almond shells ethanolic extract on hematological parameters. Where *p < 0.05, **p < 0.001 and ***p < 0.0001.

Almond shells ethanolic extract showed slight decrease in WBCs and an increase in RBCs and HB level when compared to CCl_4_ intoxicated group, as shown in graph Fig. 3. Treatment of silymarin, 200 mg/kg, 400 mg/kg and 600 mg/kg of ethanolic extract of almond shells showed a remarkable decrease while CCl_4_ treated group (group 2) showed a significant increase in WBCs (8.61 × 10^3^/Cu.mr, 18.5 × 10^3^/Cu.mr, 16.9 × 10^3^/Cu.mr and 15.2 × 10^3^/Cu.mr VS 20.6 × 10^3^/Cu.mr) with respect to control. Previous studies reported the elevated white blood cell (WBC) counts have been linked to several conditions including cardiovascular disease, infections, diabetes, liver toxicity and metabolic syndrome (MS) (Chung et al., 2016). On the other hand, almond shells ethanolic extract shows a significant recovery (p < 0.001) in RBCs than CCl_4_ treated group (7.5 × 10^6^Cu.mr, 8.3 × 10^6^/Cu.mr and 8.9 × 10^6^/Cu.mr VS 9.08 × 10^6^/Cu.mr). Similarly, Hb levels was also recovered near to control group in almond shells treated group but Hb of 200 mg/kg and 400 mg/kg was observed lower than the CCl_4_ intoxicated group and 600 mg/kg showed significant recovery of Hb level (13.2 G/dL, 14.3 G/dL and 15.2 G/dL VS 14.7 G/Dl). (See Fig. 3).

### 3.7 Histopathological examination

**Fig. 4:**
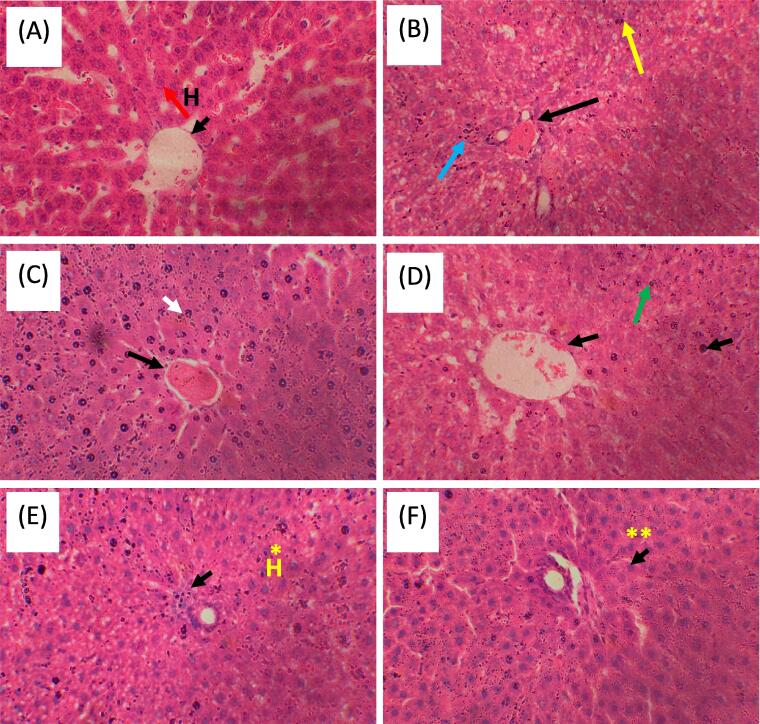
Effect of almond shell extract on liver histopathological alterations and hepatocyte apoptosis in CCl_4_-treated rats. (A) Normal Histology indication normal hepatocytes and hepatic portal vain (B) CCL_4_ treated group showing cellular apoptosis indicated with blue arrow, hepatic hemorrhage indicated with black arrow and cellular debris accumulation indicated with yellow arrow (C)Silymarin treated group highlighting normal hepatocytes (white arrow) and hepatocytic hemorrhage (black arrow) (D) 200 mg/kg ASEE treated group showing recovery of hepatocytes and reduction of apoptosis (E) 400 mg/kg ASEE treated group showing significant * recovery of hepatocytes and reduction of hemorrhage (F) 600 mg/kg ASEE treated group showing more significant ** recovery of hepatocytes very similar to control and normal hepatic portal vein.). (H&E, inserted images 40X).

The liver of dissected rats were observed by the naked eye for morphological changes and images were taken. Toxicity caused by CCl_4_ was observed by histopathological examination. Group 6 showed the least damage which was treated with 600 mg/kg of almond shells Fig. 4.

## 4 Discussion

In the present study, methanolic extract of almond shells was used for the treatment of carbon tetrachloride intoxicated rats. To check the potential of almond shells methanolic extract against CCl_4_ different parameters were analyzed, Extract decreases the level of WBCs and slightly increases the level of Hb and hemoglobin as the concentration of extract increases. Almond shells ethanolic extract showed good antioxidant activity and lowered the levels of ALP, ALT and AST, thus exhibiting hepatoprotectivity.

The hepatoprotectivity of almond oil was investigated by Jia et al. against CCl_4-_induced toxicity in rats. CCl_4_ significantly increased the levels of AST, ALP and ALT whereas almond oil significantly decreased the level of these enzymes at different concentrations. Histopathology of the liver also revealed a hepatoprotective effect in almond shell oil-treated rats (Jia et al., 2011). The same results were showen in our studies by the use of ASEE at different concentrations against CCl_4_. Carbon tetrachloride increases the levels of AST, ALP and ALT whereas ASEE lowered the level of these enzymes in a dose-dependent manner.

Ali et al. determined the antioxidant activity of almond hulls and shells methanolic extract in vitro by hydrogen peroxide, nitric oxide and superoxide radical inhibition assay. Almond hulls and shells showed powerful antioxidant activities due to the presence of triterpenoids, phenolic acids and flavonoids and thus verified our results (Ali et al., 2008). In the present study, almond shells ethanolic extract showed maximum antioxidant activity by DPPH assay at 500 µg/ml at 517 nm absorbance. Hematological parameters of almond shells ethanolic extract were also observed in the present study. ASEE increased the levels of RBCs and HB and decreased the WBCs with the level of extract concentration. Similarly, silymarin which was the positive control of our studies, showed a decrease in WBCs.

In this study, experimental animals were exposed to carbon tetrachloride to cause liver and kidney damage. Due to CCl_4_ toxicity, levels of creatinine and urea are elevated, whereas ASEE at different concentrations lowers the creatinine and urea level. Li et al. also showed a decrease in kidney biomarkers in their hepatoprotective study on rats, thus this study also favors our results (Li et al., 2013).

## 5 Conclusion

This study was designed to investigate the hepatoprotective activity of food wastes. For this purpose, twenty food wastes of fruits and vegetables were screened for antioxidant activity and found almond shells to a highly potent with IC_50_: 15.4 µg/ml. Then *in vivo* model was developed for the assessment of hepatoprotectivity of almond shell ethanolic extract against CCl_4_-induced toxicity rat model. The serum analysis and histopathological data indicated the hepatoprotective effect of almond shells. To the best of our knowledge this is the first study on hepatoprotectivity of almond shell ethanolic extract against CCl4 induced toxicity. Furthermore, pre-clinical studies are recommended before applying to drug development for clinical uses.

## Declaration of Competing Interest

The authors declare that they have no known competing financial interests or personal relationships that could have appeared to influence the work reported in this paper.
